# *Aloe vera* Oil-Added Agar Gelatin Edible Films for Kashar Cheese Packaging

**DOI:** 10.1021/acsomega.3c00147

**Published:** 2023-05-18

**Authors:** Isılay Isık, Hande Yenipazar, Ayse Saygun, Nese Sahin Yesilcubuk, Esra Ozkan Zayim, Huceste Catalgil Giz

**Affiliations:** † Department of Food Engineering, Faculty of Chemical and Metallurgical Engineering, Istanbul Technical University, Maslak, Istanbul TR 34469, Türkiye; ‡ Department of Physics Engineering, Faculty of Science and Letters, Istanbul Technical University, Maslak, Istanbul TR 34469, Türkiye; § Department of Chemistry, Faculty of Science and Letters, Istanbul Technical University, Maslak, Istanbul TR 34469, Türkiye

## Abstract

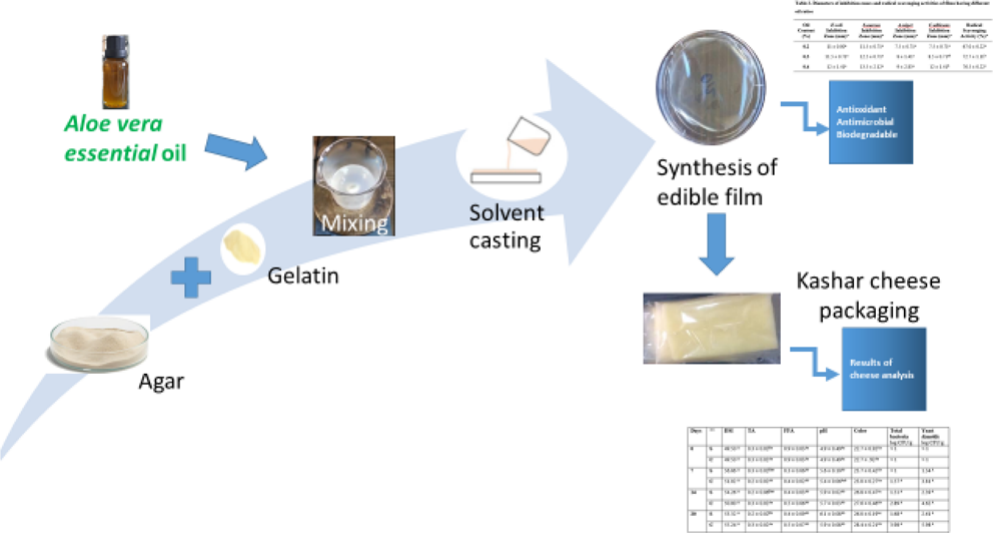

In recent years, there has been a growing interest in edible and biodegradable films due to their sustainability, environmental friendliness, and their functionality. In this work, *Aloe vera* oil-added agar–gelatin films were prepared and characterized in terms of water content, degree of swelling, water solubility, antioxidant activity, and antimicrobial activity. The possibility of using these edible films for Kashar cheese packaging during cold storage was investigated. Physical, chemical, and microbiological properties of the packaged cheese samples were examined for 20 days of cold storage at 4 °C. *A. vera* oil-added films were found to have antibacterial activity against *Escherichia coli* and *Staphylococcus aureus* and antifungal activities against *Aspergillus niger* and *Candida albicans*. *A. vera* oil-added films showed high antioxidant activities, increasing with the increasing *A. vera* oil percentage in the formulation. The current study showed that at the end of 20 days of storage period, bacterial growth in *A. vera* oil-incorporated film-covered samples was 2.30 log CFU/g lower than the control samples, and the amount of yeast and mold in *A. vera* oil-added film-covered samples was 3.37 log CFU/g lower than control samples. This shows the efficiency of *A. vera* oil-incorporated agar–gelatin films during the refrigerated storage period. Our data evidenced the positive effect of *A. vera* oil-added agar–gelatin films on Kashar cheese packaging as an innovative and sustainable technique to maintain cheese quality and prevent food loss during storage.

## 1 Introduction

In recent years, there has been a growing interest in edible and biodegradable films, which can be promising for food packaging applications. Hydrogels are polymeric networks that can absorb aqueous solutions with their hydrophilic properties and maintain their structure without dissolving. Hydrogel films can be manufactured with natural and synthetic polymers. Polysaccharide derivatives (cellulose-based chitin–chitosan, pectin, starch-based potato, wheat, alginate, agar, carob, and guar), protein derivatives (gelatin, collagen, wheat protein, milk proteins, corn zein, and soy protein), and lipid sources (acetylated monoglycerides, natural waxes, and various fatty compounds) are examples of natural polymers, whereas polyvinyl alcohol and polyvinyl pyrrolidone are synthetic polymers.^1,2^

Edible biopolymers are innovative alternatives and mostly preferred in the food industry due to their biocompatible, biodegradable, environmentally friendly properties.^3−5^ Studies suggest that edible coatings have been playing a vital role in improving the shelf life and the quality of the products such as meat, poultry, seafood, ready-to-eat foods, fresh fruits, and vegetables.^6,7^ Essential oils and *Aloe vera* gel are also widely studied as natural components of edible coatings.^8^

Kashar cheese is one of the most consumed cheese types in Turkey and can be sub-grouped into semi-hard and pasta filata type cheeses.^9^ Diverse versions of this cheese exist in distinct areas, such as Kaskaval Preslav in the Balkan region, Kashkaval in Russia, and Caciocavallo in Italy.^10^ Mostly, Kashar cheeses are packaged with synthetic packaging materials like polyethylene in modified atmosphere. Cheese is very susceptible to microbial and chemical spoilage.^11^ One of the main causes of cheese losses is microbial growth due to bacterial and fungal strains.^12^ Fungi growth is one of the most important factors that affect the quality and storage conditions of Kashar cheese. A recent study showed that Kashar cheese is prone to microbiological and oxidative deterioration during storage due to the amount of casein and water-soluble nitrogen in the cheese, which shortens the shelf life of the product.^13^ Also, high moisture loss is another important problem causing a decrease in the quality of cheese and leading to undesired organoleptic properties. To solve these problems, edible film packaging and coatings are promising materials.^14^

Elgayyar et al. (2001) evaluated the antimicrobial efficiency of oregano essential oil against *Escherichia coli* O157:H7, *Staphylococcus aureus,* and *L. monocytogenes* and proposed that they can be used as an alternative to conventional antimicrobial additives in foods.^15^ Fajardo et al. (2010) studied the shelf life of Saloio cheese, which was coated with natamycin-added chitosan edible films.^16^ There was a significant decrease in the mesophilic and psychrophilic bacteria in chitosan-whey protein-covered Ricotta cheeses at the end of the storage period.^17^ Kashar cheese samples were coated with thyme and clove oil-containing edible films.^18^ Antimicrobial effects of these films against *E. coli*, *S. aureus,* and *L. monocytogenes* were evaluated. It was found that thyme and clove essential oils prevent microbial growth on Kashar cheese and thyme oil had a slightly higher activity than clove oil.^18^ Artiga-Artigas et al. (2017) used 2.0% oregano essential oil and reduced the growth of *S. aureus* below 4.6 log CFU/g compared to the control samples (6 log CFU/g) after storing the low-fat cheese at +4 °C for 15 days.^19^ Mezhoudi et al. (2022) studied the effect of the film made from gray triggerfish gelatin and enriched with *Moringa oleifera* ethanolic extract in wrapping ricotta cheese.^20^ They found that this edible film reduced microbiological spoilage and preserved the physical quality of ricotta cheese.^20^ There is another research studying fish gelatin and grape seed extract on moisture state, microbiota composition, and quality of chilled sea bass fillets. Zhao et al. (2021) showed a synergistic preservative effect of fish gelatin and grape seed extract, and this combination inhibited the growth of *Pseudomonas*, *Aeromonas*, *and Shewanella*; also, the total viable count and spoilage bacteria were reduced by >1 log CFU/g as compared to the control.^21^ These works showed that the shelf life of cheeses can be extended and quality can be improved with the use of edible films having antimicrobial and antifungal properties.

In this work, *A. vera* oil-added agar–gelatin films were prepared and applied to Kashar cheese packaging.

Agar is a polysaccharide derived from red algae, which produces transparent films with high tensile strengths and elongation at break values. Due to their crosslinked nature, agar films are not water-soluble up to 90 °C. Pure agar films are hard and tough and vulnerable to microbial attacks. For this reason, agar films are usually applied in the compounded form. Recently, agar-locust bean gum and agar-salep films were prepared, and physical properties and antimicrobial properties were investigated.^22^

Gelatin is one of the animal-based proteins used for thickening and texturizing the foods in food industry, due to its good gelatinization properties. It has a strong film-forming feature and shows a protective effect against drying, light, and oxygen.^23,24^ It is water-soluble and also produces transparent films at room temperature;^4^ however, gelatin films do not have antimicrobial effects.^25,26^

Agar–gelatin film production was chosen; however, they are prone to microbial attacks. To solve this problem, *A. vera* oil was added to the films as an antimicrobial agent.

The long-lived, pea-green-colored plant *Aloe* is a member of the *Asphodelaceae* family and has been used in the food industry as an ingredient in functional foods and in health products.^27^*A. vera* essential oil is composed of especially 1,8 dihydroxyanthraquinone derivatives and their glycosides and has been used as an antibacterial and antioxidant agent in many different medical applications in traditional medicine.^28^ It is now well established from a variety of studies that the bioactive components of *A. vera* (which contains approx. 98.5% water; the remaining 1–1.5% solid material consists of a range of compounds including water-soluble and fat-soluble vitamins, minerals, enzymes, polysaccharides, phenolic compounds, and organic acids) have antifungal, antiseptic, antibacterial, anti-inflammatory, and antioxidant properties.^29−32^ Furthermore, the antimicrobial effects of *A. vera* are attributed to the natural anthraquinones of this plant.^33^

In a comprehensive study on the antibacterial and antioxidant properties of *A. vera* leaf gel, which is extracted with different solvents such as distilled water, ethanol, and acetone, it was conclusively shown that *E. coli* was found to be more sensitive than *S. aureus* to acetone extract and ethanol extract had no impact on *E. coli* and minor impact on *S. aureus*.^34^ In the review of Misir et al. (2014), the preparation, properties, and potential application of *A. vera* gel coatings for enhancing the postharvest life and quality of different types of fruits were described.^35^

To the best of our knowledge, the current study is the first research reporting preparation of *A. vera* oil-added agar–gelatin edible films and application of these films for the packaging of Kashar cheese in order to improve the shelf life and quality during cold storage.

During storage for 20 days at 4 °C, physical, chemical, and microbiological properties of Kashar cheese were investigated and compared with a polyethylene cling film wrapped control group.

## 2 Materials and Methods

### 2.1 Materials

Agar and gelatin were purchased from Sigma-Aldrich (St. Louis, MO, USA). *A. vera* essential oil (obtained by steam distillation from leaves) was purchased from Defne Doğa (Antalya, Turkey). Distilled water was used during film preparations. DPPH (2,2-diphenyl-1- picril-hydrazyl) solution and ethanol were obtained from Merck (Darmstadt, Germany). Gram-positive *S. aureus* (*S. aureus*) ATCC 6538 and Gram-negative *E. coli* (*E. coli*) ATCC 43888 bacterial strains were both cultured in nutrient broth and nutrient agar (Merck, Darmstadt, Germany). The fungus, *Aspergillus niger* (*A. niger*) ATCC 16404, was kept on Potato dextrose agar (Fluka) at +4 °C. *Candida albicans* (*C. albicans*) was kept on Sabouraud dextrose agar (Merck, Darmstadt, Germany) media. Samples of Kashar cheese were obtained from a local market in Istanbul.

### 2.2 Film Preparation

Edible films were prepared with agar (75%) (*w*_agar_/*w*_tot)_ and gelatin (25%) (*w*_gel_/*w*_tot_) with different concentrations [0.2, 0.3, 0.4, 1, and 2% (*w*_oil_/*w*_biopolymer_)] of *A. vera* oil by the solvent casting method. 0.75 g of agar and 0.25 g of gelatin and *A. vera* oil were added to 100 mL of distilled water and heated up to 90 °C and homogenized by Ultra Turrax T18 (IKA, Germany). 30 g of prepared solutions was added in Petri dishes (8.5 cm diameter) and dried overnight at 55 °C.

### 2.3 Characterization of Films

#### 2.3.1 Water Content, Degree of Swelling, and Water Solubility

To measure the water content, films were cut into 2 × 2 cm strips and left to dry at room temperature for 24 h (Wo). In order to get the initial dry weight (W1) of the films, they were dried at 75 °C for 24 h. The films were placed on Petri dishes, in which there was 30 mL of distilled water, and stored there for a day. After removing the surface water by using a paper towel, samples were weighed (W2). The final dry weight (W3) was obtained by drying at 30 °C the remaining films for another 24 h.^36^ Measurements were repeated three times.

The data were collected to calculate the water content, degree of swelling, and water solubility with the following equations according to Shiku et al.^37^ and Nie et al.^38^
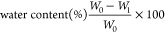
1
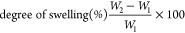
2
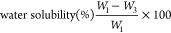
3

#### 2.3.2 Antimicrobial Activity of the Films

The agar disc diffusion method was used to measure the antimicrobial activities of films. In order to determine the antibacterial activity, *E. coli*, a Gram-negative bacteria, and *S. aureus*, a Gram-positive bacteria, were used. *C. albicans* and *A. niger* were used to investigate antifungal activity of the films.

Overnight cultures were used in the disc diffusion method.^39,40^ Test samples (films) were sterilized under UV light for 30 min. For testing the antimicrobial activity of the film samples, two pieces of 0.6 cm diameter circular films were aseptically cut and placed in the Petri dish, where 0.1 mL of bacterial culture has been previously added. *E. coli* and *S. aureus* were incubated at 37 °C for 24 h, as *C. albicans* and *A. niger* were incubated for 3 days at 25 °C.

#### 2.3.3 Antioxidant Activity of the Films

The method described by Yang et al. (2010)^41^ for DPPH (2,2-diphenyl-1-picril-hydrazyl) analysis has been followed.

The principle of the DPPH radical scavenging method is the reduction of the DPPH free radical, the antioxidants reacting with these stable DPPH radicals and converting the radicals into 1,1-diphenyl 2-picril hydrazine. The ability to remove stable DPPH radicals is measured by a decrease in absorbance. DPPH at an amount of 3.943 mg was dissolved in 100 mL of ethanol (100%) to obtain 0.1 mmol/L DPPH solution. This solution was then diluted to obtain 0.01 mmol/L DPPH solution. The film sample of 25 mg was dissolved in 8 mL of distilled water. DPPH solution (3.9 mL of 0.01 mmol/L) was added to each sample and kept at room temperature in a dark environment for 30 min. Then, absorbances were measured using a spectrophotometer (LR45227 Thermo Fischer Scientific, USA) at 517 nm.^33^ Ethanol was used as a control sample.

Radical scavenging activity was calculated using the following equation
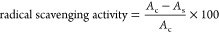
4where *A*_c_ and *A*_s_ indicate control and sample results, respectively.

### 2.4 Edible Film Application to Kashar Cheese

Kashar cheese samples were sliced at 8 mm thickness and individually wrapped with *A. vera* oil-containing edible films. Control Kashar cheese samples were wrapped with polyethylene cling film and stored at +4 °C in a separate refrigerator to prevent any cross-contamination. Samples were analyzed on days 0, 7, 14, and 20. Chemical, physical, and microbiological experiments were conducted at these time intervals.

### 2.5 Analysis of Kashar Cheese Samples

#### 2.5.1 Determination of Total Dry Matter, Titratable Acidity, Free Fatty Acid, pH and Color Analysis

Determination of the total solids content was performed by using reference method ISO 5534:2004. 3 g of the sample was mixed with 20 g of sea salt, and the mixture was left to dry in the oven at 102 °C. Total dry matter content (%) was calculated using the following equation
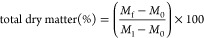
5

In the equation given above, *M*_f_ is the weight of the sample plus the plate after drying (g), *M*_1_ is the sample plus the weight of the plate before drying (g), and M_0_ is the tare weight (g).

Titratable acidity of Kashar cheese was analyzed by using the TSE 591 official method. 25 g of the sample was titrated with 0.1 M sodium hydroxide solution. Phenolphthalein was used as an indicator. Acidity (%) of Kashar cheese was calculated by the titrimetric method over the amount of sodium hydroxide spent in titration with the following equation
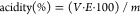
6where *V* is the amount of 0.1 M NaOH (mL) spent in titration, *E* is the equivalent acidity value (g) of 1 mL 0.1 M NaOH, and *m* is the weight of the titrated sample.

AOCS official method Ca 5a—40 was used to determine the free fatty acid content of Kashar cheese. 10 g of the sample was mixed with 50 mL of ethanol/diethyl ether (1:1 *v*/*v*). The mixture was titrated with 0.1 N sodium hydroxide after adding three drops of phenolphthalein. The percentage of the free fatty acid was calculated by eq 7.
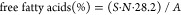
7where *S* is the amount of NaOH spent (mL), *N* is the normality of sodium hydroxide, and *A* is the weight (g) of the sample.

The pH values of Kashar cheese samples were measured using a pH meter (Testo 205, Germany).

The color of the covered Kashar samples was measured from the surface reflection by using a Minolta CR-400 colorimeter device (Minolta Corp, Ramsey, NJ, USA). The color parameters, which coordinate the color tone, are L*, a*, and b*. L* measures lightness, a* measures the green—red difference, and b* measures the blue—yellow difference.

#### 2.5.2 Microbiological Analysis of Cheese, Total Aerobic Mesophilic Bacterial Count, and Yeast and Mold Count

Tryptic soy agar (TSA) was used as a growth medium for the total aerobic mesophilic bacterial counts. Kashar cheese (10 g) was homogenized for 1.5 min in the stomacher with 90 mL of peptone water. Samples were diluted to 10^–3^, and each dilution was inoculated in Petri dish using the spread plate method. The Petri dishes are incubated at 37 °C for 24–48 h.^42^

Dichloran Rose Bengal Chloramphenicol was used as agar medium for yeast and mold analysis. Kashar cheese (10 g) was homogenized for 1.5 min in the stomacher with 90 mL of peptone water. Samples were diluted to 10^–3^, and each dilution was inoculated in Petri dish using the spread plate method. The Petri dishes are incubated at 25 °C for 3 days for mold count and 5 days for yeast count.^43^

### 2.6 Statistical Analysis

Each experiment was performed three times, and the average result and standard deviation are given by the MINITAB software (version 16.1.0) for one factor analysis of variance (ANOVA) test and *p* < 0.05 (95%) of the probability value is quoted. The difference between the results was determined using the Tukey test at a 95% confidence level (*p* < 0.05).

## 3 Results and Discussion

### 3.1 Film Preparation

Edible films that have been left to dry for 24 h were taken out from the drying oven, and it was observed that films having 1 and 2% of *A. vera* oil had oily surfaces, which were not further used, whereas 0.2, 0.3, and 0.4% of *A. vera* oil-containing films did not have surface oil; therefore, these films were selected for further analysis.

### 3.2 Characterization of Films

#### 3.2.1 Water Content, Degree of Swelling, and Water Solubility

Water content, degree of swelling, and water solubility are important properties for the suitability of the films as packaging for food materials. For example, if an edible film is used as a coating material for high moisture foods, the film should have a low water solubility.^44^

Water content, degree of swelling, and water solubility results of the film samples (containing 0.2, 0.3, and 0.4% *A. vera* oil) and the control group (without *A. vera* oil) are given in Table 1.

**Table 1:** Water Content, Degree of Swelling, and Water Solubility of Edible Films^b^

oil content (%)	water content (%)^a^	degree of swelling (%)^a^	water solubility (%)^a^
0	12.4 ± 1.64^a^	325 ± 14^ab^	12.5 ± 1.59^a^
0.2	11.7 ± 1.51^a^	292 ± 14^a^	15.5 ± 1.00^ab^
0.3	11.4 ± 0.55^a^	347 ± 77^ab^	18.5 ± 2.84^b^
0.4	12.8 ± 0.90^a^	423 ± 28^b^	18.2 ± 2.37^b^

a Results are given as mean ± standard deviation of three independent experiments.

b ^a,b^Differences between results are indicated with lettering according to the statistical experiments (*p* < 0.05).

Although water contents of *A. vera* oil films did not differ significantly, degree of swelling and water solubility values of films slightly increased as the *A. vera* oil percentage was increased.

#### 3.2.2 Antimicrobial Activity of Films

Pure agar^22^ and pure gelatin^45^ films does not have an antimicrobial effect. Addition of *A. vera* oil supplied the necessary antimicrobial properties. Table 2 shows the diameters of inhibition zones. It can be seen from Table 2 that antimicrobial activity increased for all microorganisms when oil content of the films increased. The largest diameter (11.5 ± 0.71 mm) was measured with 0.4% oil-containing film, against *S. aureus*, whereas diameters of the narrowest zones (7.5 ± 0.71 mm) were against *A. niger* and *C. albicans* with 0.2% oil-containing films. According to these results, *S. aureus* was more susceptible to inhibition due to *A. vera* oil according to the disc diffusion assay method. Moreover, *A. vera* oil was found to be more effective against bacteria than yeast and mold.

**Table 2:** Diameters of Inhibition Zones and Radical Scavenging Activities of Films Having Different Oil Ratios^b^

oil content (%)	*E. coli* inhibition zone (mm)^a^	*S. aureus* inhibition zone (mm)^a^	*A. niger* inhibition zone (mm)^a^	*C. albicans* inhibition zone (mm)^a^	radical scavenging activity (%)^a^
0.2	11 ± 0.00^a^	11.5 ± 0.71^a^	7.5 ± 0.71^a^	7.5 ± 0.71^a^	67.0 ± 0.22^a^
0.3	11.5 ± 0.71^a^	12.5 ± 0.71^a^	8 ± 1.41^a^	8.5 ± 0.71^ab^	72.7 ± 1.11^b^
0.4	12 ± 1.41^a^	13.5 ± 2.12^a^	9 ± 2.83^a^	12 ± 1.41^b^	76.5 ± 0.22^c^

a Results are given as mean ± standard deviation of three independent experiments.

b ^a,b,c^Differences between results are indicated with lettering according to the statistical experiments (*p* < 0.05).

#### 3.2.3 Antioxidant Activity of Films

Radical scavenging activities were calculated according to the absorbance values, and the results are given in Table 2. It was observed that the higher the oil content of the films had, the higher their antioxidant activity.

In order to determine the most suitable edible film formulation, antimicrobial and antioxidant results were taken into consideration. Film having 0.4% of *A. vera* oil was more effective on microbial inhibition and had a higher antioxidant potential. Thus, 0.4% of *A. vera* oil-containing edible film was considered as a packaging material for Kashar cheese samples in the further applications.

According to a research, antioxidant activity of crude *A. vera* gel was found to be (17.11 ± 1.30%) by DPPH assay, lower than the results of *A. vera* oil, obtained in this study.^46^ The extraction method and gel or oil forms of *A. vera* affect the radical scavenging activity. In another study, methanol extraction was found to show the highest DPPH inhibition, and methanol was found to be more efficient than water in cell walls due to nonpolar character and causing active ingredients to be released from cells.^47^ In particular, fiber content of the sample decreases the scavenging activity and thus antioxidant property as well.^48^

Higher antioxidant activity may be explained by higher polyphenol content and lipophilic properties of *A. vera* oil. Similarly, Feng et al. (2020) concluded that tocopherol nanoemulsions retarded lipid oxidation and improved the quality of the fish sausages due to higher polyunsaturated fatty acid and lower peroxide value of tocopherol.^49^

### 3.3 Cheese Analysis

In this part of the study, the physico-chemical, color and microbiological analysis of Kashar cheeses packaged with *A. vera* oil-added agar–gelatin film and polyethylene cling film (control) were compared on the 0th, 7th, 14th, and 20th days of refrigerated storage.

#### 3.3.1 Total Dry Matter, Titratable Acidity, Free Fatty Acid, pH, and Color Results of Cheese

The dry matter content of samples are given in Table 3. As can be seen, total dry matter values of control and the edible film wrapped cheese samples generally varied between 49.50 and 56.06%. The dry matter contents of the control samples increased during the storage period. However, the change in total solids content of the control and the cheese samples were not considered statistically significant. In a recent study, Civelek and Cagri-Mehmetoglu^50^ had established similar results. They concluded that during storage, the solids content of vacuum-packaged cheese samples increased 0.6–1.1% from moisture loss. Treating with different coatings did not significantly affect cheese solid contents.^50^

**Table 3:** Cheese Analysis Results^a^^,^^b^

days	c	DM	TA	FFA	pH	color	total bacteria log CFU/g	yeast and log CFU/g
0	**S**	49.50^A^	0.3 ± 0.02^Ba^	0.9 ± 0.03^Aa^	4.9 ± 0.49^Aa^	22.7 ± 0.92^Aa^	<1	<1
	**C**	49.50^A^	0.3 ± 0.02^Aa^	0.9 ± 0.03^Aa^	4.9 ± 0.49^Aa^	22.7 ± 0.92^Aa^	<1	<1
7	**S**	56.06^A^	0.3 ± 0.02^Bab^	0.3 ± 0.06^Ab^	5.6 ± 0.10^Ab^	23.7 ± 0.42^Ab^	<1	1.34^A^
	**C**	51.02^A^	0.2 ± 0.02^Ab^	0.4 ± 0.02^Ab^	5.4 ± 0.06^Aab^	23.0 ± 0.27^Aa^	1.57^A^	3.81^A^
14	**S**	54.26^A^	0.2 ± 0.06^Bab^	0.4 ± 0.03^Ab^	5.9 ± 0.02^Ab^	26.8 ± 0.47^Ac^	1.51^A^	2.39^A^
	**C**	50.00^A^	0.3 ± 0.02^Aa^	0.5 ± 0.06^Ab^	5.7 ± 0.03^Ab^	27.6 ± 0.46^Ab^	2.89^A^	4.62^A^
20	**S**	53.32^A^	0.2 ± 0.02^Bb^	0.4 ± 0.09^Ab^	6.1 ± 0.08^Ab^	26.0 ± 0.19^Ac^	1.68^A^	2.61^A^
	**C**	55.24^A^	0.3 ± 0.02^Aa^	0.5 ± 0.07^Ab^	5.9 ± 0.08^Ab^	28.4 ± 0.21^Ab^	3.98^A^	5.98^A^

a DM: dry matter, TA: titratable acidity, FFA: free fatty acids.

b Capital letters indicate statistical significance between control and sample groups (*p >* 0.05). Small letters define the statistical difference in each group (*p >* 0.05). For color, the reported values are relative to the b* value.

c C: control; S: sample.

The average results of titratable acidity of samples are given in Table 3. Titratable acidity of the control group was significantly different according to ANOVA results. All these results are given in terms of lactic acid, which was formed as a result of breakdown of lactose.^51^ The titratable acidity values of the samples varied between 0.2 and 0.3% during storage and are shown in Table 3.

According to the Table 3, free fatty acid content of edible film-wrapped cheese samples was slightly lower than that of the control group, but the difference was not significant. Free fatty acidity of cheese samples was found to vary between 0.3 and 0.9% during storage.

The pH value of both the samples and the control group increased, from 4.9 to 5.9 and 6.1, respectively, on the 20th day during the storage period, as can be seen in Table 3.

The color of the samples was influenced by the increase in the b* value, which indicates the prevalence of yellow color in cheese.^19^ A significant increase of the b* value was observed throughout the storage, without any significant differences between the edible film covered samples and the control samples (Table 3). As for the control samples, the b* value varied between 22.7 and 28.4.

#### 3.3.2 Microbiological Analysis of Cheese Samples

##### 3.3.2.1 Total Aerobic Mesophilic Bacterial Counts

According to the results of the total aerobic mesophilic bacterial counts shown in Table 3, bacterial growth increased in the control samples and reached the highest value of 3.98 log CFU/g at the end of the storage period. Bacterial growth in the edible film-covered samples were found to be 1.68 log CFU/g, which was more than 200 times lower than the control samples (*p* < 0.05). Alginate nanoemulsion films containing at least 2.0% (*w*/*w*) oregano oil *S. aureus* population log CFU/g value was 4.6 instead of 6.0 in control sample after 15 days.^19^ In our study, total bacterial count log CFU/g value was 1.68 instead of 3.98 after 20 days of storage using *A. vera* oil.

##### 3.3.2.2 Yeast and Mold Counts

Fungi analysis results are given in Table 3 show that *A. vera* oil had a significant inhibitory effect on mold and yeast growth in edible film-covered Kashar cheese. On the 20th day of storage, the amount of yeast and mold in the control samples had reached the highest value of 5.98 log CFU/g. Maximum fungi growth was observed as 2.61 log CFU/g in edible film-covered cheese samples.

Tomar and Akarca (2019) used locust bean gum films containing sage, rosemary, oregano, cinnamon, and ginger essential oils and found that cinnamon has the highest antimicrobial activity as 4.02 logCFU/g after 21 days.^52^ In another study conducted by Fajardo et al. (2010), natamycin-containing chitosan-coated Saloio cheese samples presented a decrease on molds/yeasts of 1.1 log CFU/g compared to control after 27 days of storage.^16^ In this study, 3.37 logCFU/g decrease was observed using *A. vera* oil after 20 days storage of cheese sample. Azhdari and Moradi (2022) studied the coating of mozzarella cheese with natamycin and concluded that the coating with natamycin at 0.05 and 0.5% represented a 0.6 and 0.9 log cycle reduction in yeast-mold populations, respectively. Based on the total mesophilic counts, the control samples reached the 7 log CFU/g on day 4, indicating a 4 day shelf life of HMMC, while in high moisture mozzarella cheese coated with and without natamycin, this limit was reached on the 8th day of storage.^11^

## 4 Conclusions

Edible films incorporated with *A. vera* oil showed antimicrobial activity against all the microorganisms that were investigated in this study (*E. coli*, *S. aureus*, *A. niger,* and *C. albicans*). *A. vera* oil was found to have the highest antimicrobial activity against the microorganism *S. aureus*. *A. vera* oil with 0.4% of concentration was used in films which has a radical scavenging activity of 76.5%.

To our knowledge, the current study is the first research describing the quality changes of cheese samples wrapped with *A. vera* oil-added agar–gelatin films during the cold storage period. The current study showed that at the end of 20 days of the storage period, bacterial growth in the edible film-covered samples was 1.68 log CFU/g lower than the control samples (3.98 log CFU/g). The amount of yeast and mold was 2.61 log CFU/g in edible film-covered samples versus 5.98 log CFU/g in the control samples. *A. vera* oil has antifungal and antibacterial properties, which provide a defensive barrier against microbial contamination of cheese samples.

Considering the results of this study, the present study indicates that *A. vera* oil-added edible film-covered Kashar cheese exhibited better quality parameters during storage and prevented microbial growth compared to the control cheese samples, thereby providing a good alternative for the future biodegradable food packaging applications.
